# Intervention measures for stigma in HIV patients: a scoping review of randomized controlled trials

**DOI:** 10.3389/fpubh.2025.1655870

**Published:** 2025-11-05

**Authors:** Mingrui Zhang, Hongxu Zhu, Yi Xu, Xiahui Li, Kaihan Yang, Bei Niu, Xue Wang

**Affiliations:** ^1^School of Basic Medical Sciences, Chengdu University, Chengdu, China; ^2^School of Nursing, Chengdu University, Chengdu, China

**Keywords:** HIV, AIDS, stigma, intervention, RCT, scoping review

## Abstract

**Background:**

Individuals infected with HIV experience significant stigma, and since this stigma can severely impact their quality of life, it is essential to implement interventions aimed at reducing the stigma faced by this population.

**Objective:**

To summarize and analyze the core components and effectiveness of interventions targeting stigma among people living with HIV. Methods A scoping review methodology was employed to search the PubMed, Web of Science, Embase, Cochrane, and Scopus databases from their inception dates up to March 14, 2025. The included studies were categorized and analyzed.

**Results:**

A total of 39 articles were included in this review. Interventions addressing HIV-related stigma were conducted among various populations including pregnant women, older adults, adolescents, and sexual minority groups. Intervention contents included cognitive behavioral therapy, motivational interviewing, information-motivation-behavioral skills intervention, among others. There was significant variation in the frequency and duration of stigma interventions. Outcome measures used across studies included internalized stigma, externalized HIV Stigma, enacted stigma.

**Conclusion:**

The findings indicate that most interventions demonstrate varying degrees of effectiveness in reducing HIV-related stigma, with CBT-based approaches potentially being more effective, although standardization and longer follow-up periods are required. However, the differences in effectiveness across different populations, intervention content, implementation forms, and follow-up durations suggest that current stigma reduction strategies for AIDS still have room for optimization.

## 1 Introduction

According to the Joint United Nations Programme on HIV/AIDS, by the end of 2023, there were approximately 39.9 million people worldwide living with HIV infection/AIDS, among whom 1.3 million were newly diagnosed cases in that year, and 30.7 million individuals were receiving antiviral treatment (1). With continuous advancements and effective implementation of antiretroviral therapy, the prognosis for people living with HIV has improved significantly, and their life expectancy has increased. In 2016, LAZARUS et al. proposed a new goal: ensuring that 90% of those achieving viral suppression attain good quality of life (2). This objective highlights that as survival duration extends for people living with HIV, improving their quality of life has become a key issue in global AIDS prevention and control efforts. Quality of life is a multidimensional concept encompassing physical health, psychological well-being, social relationships, and environmental adaptation. Among these dimensions, stigma stands out as one of the most significant factors affecting the quality of life of people living with HIV.

The word “stigma” originated from ancient Greece, referring to the mark left by a hot iron on prisoners, as well as to a stain or blemish identified by moral standards as abnormal or bad. Goffman pointed out that shame is a notable, marked difference (3), which makes various forms of discrimination possible, ultimately depriving individuals/groups of full social acceptance, reducing personal opportunities (4), and intensifying social inequality. In people living with HIV, HIV-related stigma mainly includes enacted stigma, anticipated stigma, and internalized stigma (5). Enacted stigma refers to actual experiences of prejudice, discrimination, and exclusion described by stigmatized individuals (6); anticipated stigma involves expectations of prejudicial reactions from others upon learning of someone’s HIV status (7); internalized stigma occurs when members of stigmatized groups accept negative societal beliefs and feelings about themselves and experience social devaluation associated with their stigmatized status (8). Stigma not only harms the mental health of people living with HIV but may also hinder disease prevention and treatment as well as social integration. At the psychological level, stigma easily leads to depression, anxiety, or self-denial among infected individuals, and even refusal of testing or delayed treatment due to fear of discrimination, seriously affecting adherence to antiviral therapy (9). Regarding social relationships, infected individuals may experience intensified loneliness and financial hardship due to family rejection, workplace discrimination, or social isolation. Furthermore, avoidance of treatment can further affect their management of personal health, creating a vicious cycle. Particularly for women, sexual minorities, drug users, and other vulnerable populations, multiple stigmas are more likely to add to the low quality of their life (10).

To alleviate stigma among people living with HIV and improve their quality of life, it is urgent to explore effective intervention measures. Existing reviews have addressed some aspects of HIV-related stigma; however, limitations remain. First, although various statistical methods have been included in previous studies, the methodological quality of these studies varies (11); second, the study locations were limited to specific regions, lacking generalizability (12); third, the populations studied were restricted to specific subgroups, limiting the transferability of findings (13). Therefore, it remains unclear whether the reported stigma-reduction interventions can be extended to other HIV populations and yield similar effects. Although several reviews have summarized interventions to combat HIV stigma and identified significant heterogeneity in interventions, controls, and outcomes (14), few reviews specifically focus on randomized controlled trials—the most robust form of evidence.

Given the current fragmented evidence and the lack of a comprehensive focus on rigorously evaluated trials, we conducted a scoping review of randomized controlled trials targeting HIV-related stigma, systematically organizing RCT-based interventions for HIV-related stigma worldwide. The aim is to address issues such as interventions to reduce stigma among HIV-infected individuals, their effectiveness, and recommendations for future research.

## 2 Define the scope of the review objectives

This scoping review aims to determine the scope and content of existing literature regarding stigma among people living with HIV, and to provide guiding recommendations for future research and practice. Specifically, this review aims to present a conceptual mapping of the content, format, indicators, stigma assessment tools, and outcomes of interventions addressing HIV-related stigma.

## 3 Inclusion criteria

The detailed eligibility criteria for the study were determined using the PICOS framework:

(1) Participants: Participants included in the study must be individuals infected with HIV, without restrictions on race, age, nationality, etc.;
(2) Intervention: The experimental group received any intervention that can reduce HIV-related stigma;
(3) Control: The control group received standard treatment or care, routine education, or is placed on a waiting list;
(4) Outcomes: The study outcomes must include HIV-related stigma, encompassing internalized HIV stigma, externalized HIV stigma, anticipated HIV stigma, and enacted HIV stigma;
(5) Design: The study must be a randomized controlled trial (RCT).

## 4 Methods

This study was initially designed as a meta-analysis and registered in PROSPERO with the identifier CRD420250611522. However, due to significant heterogeneity among the interventions and study designs included in this review, a scoping review design was deemed more appropriate. The methods of this scoping review deviated somewhat from the original PROSPERO registration. This report follows the Preferred Reporting Items for Systematic Reviews and Meta-Analyses extension for Scoping Reviews (PRISMA-ScR) checklist and explanation (15), and is registered in OSF with the DOI https://doi.org/10.17605/OSF.IO/KT9XR.

### 4.1 Search strategy

A comprehensive search was conducted across five databases, including PubMed, Web of Science, Embase, Cochrane, and Scopus, from the establishment of the databases until March 14, 2025, using various search strategies. For example, in PubMed, a combination of Medical Subject Headings (MeSH) terms and text words were applied for the following concepts: ① HIV ② stigma ③ RCT. Grey literature, such as conference abstracts and government reports, was excluded. Additionally, reference lists of selected articles were examined to identify any additional articles or studies not captured through the database searches. Detailed information on all search strategies used can be found in the Supplementary materials.

### 4.2 Study selection

**Figure 1:**
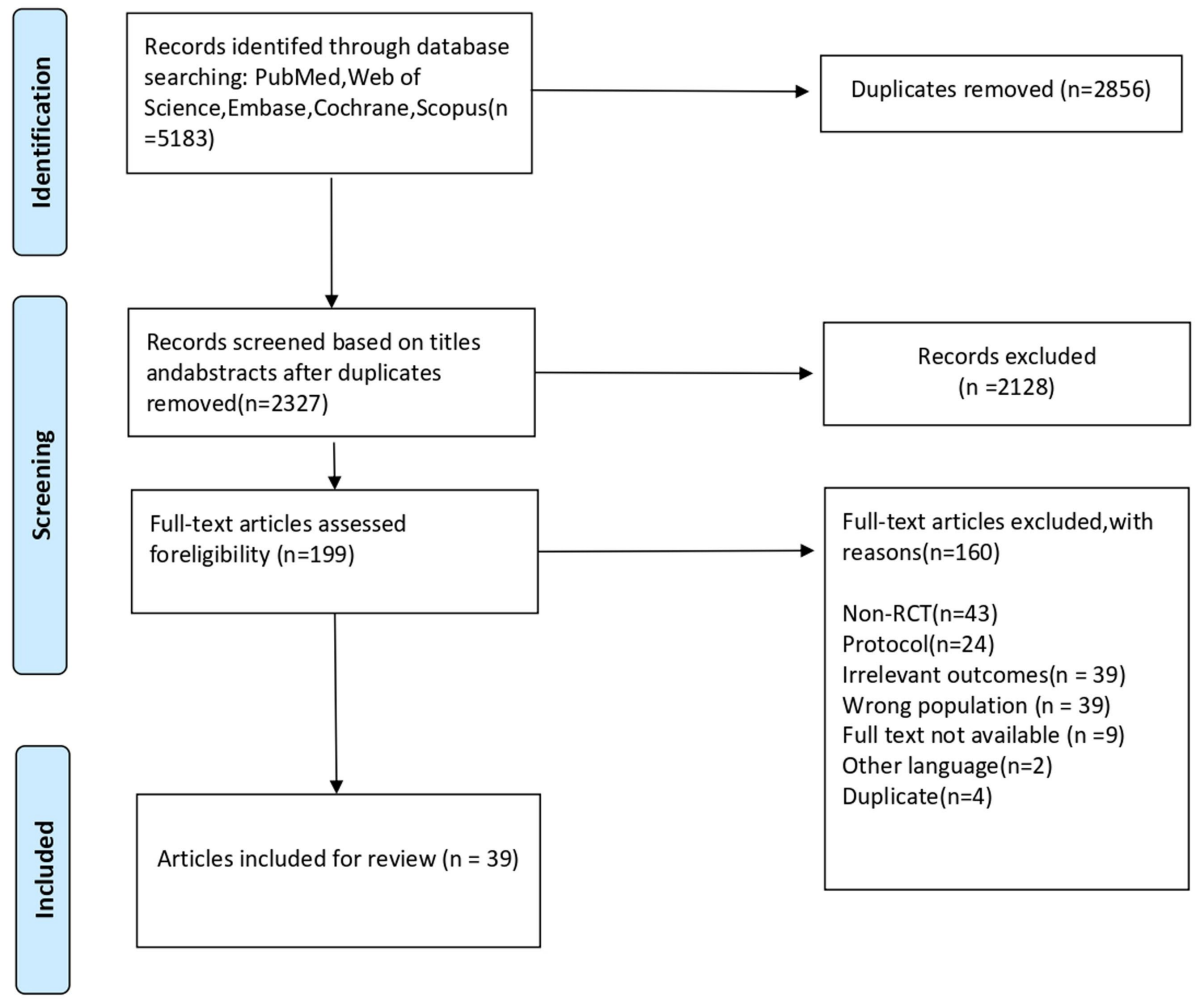
PRISMA 2009 flow diagram. From: Moher et al. (64). For more information, visit www.prisma-statement.org.

All search results were imported into the reference management program Endnote X9, and duplicates were removed. Two researchers (ZMR and ZHX) independently screened the titles and abstracts of the studies according to the inclusion criteria. We read the full texts of potentially relevant studies to select eligible articles and provided detailed reasons for exclusion. The specific screening process is shown in Figure 1. Any discrepancies during the selection process were resolved through discussion between the two researchers or consultation with a third researcher (XY).

### 4.3 Data extraction

Two researchers (ZMR and ZHX) independently extracted and recorded the data into a predesigned form. Any discrepancies were resolved through discussion or by involving a third researcher (XY). The extracted data were entered into an Excel spreadsheet for analysis. The data extraction table included the following categories: (1) general study information: first author, year, and country; (2) study type, methodology, and sample size; (3) study participants; (4) intervention content in the intervention and control groups; (5) intervention implementers, intervention format (online or offline); (6) intervention dose (frequency of interventions, duration per session, total duration of intervention, and follow-up period); (7) specific intervention content; (8) outcome measures; (9) measurement tools; (10) main outcome results of the intervention.

### 4.4 Data synthesis and analysis

The translation was conducted by two reviewers (ZMR and ZHX) after reaching a consensus through discussion. Comprehensive data from various sources led to several key observations: (1) general information of the included studies; (2) intervention content; (3) intervention format; (4) intervention implementer; (5) measures for the control group; (6) number, frequency, and duration of interventions; (7) outcome indicators and measurement tools; (8) follow-up period; (9) intervention effectiveness.

### 4.5 Critical evaluation

The objective of this study was to determine the scope of all available literature regarding interventions for HIV-related stigma; therefore, to consider the full body of available evidence, articles included in this scoping review were not subjected to formal critical appraisal (16).

## 5 Result

Systematic literature search identified 5,183 references on March 14, 2025. Thirty-nine studies were considered to meet the inclusion criteria. The characteristics of the included literature are shown in Table 1.

**Table 1:** Characteristics of included studies.

Author-or, year	Country of publication	Type of study	Sample population	Age	Intervention in the experimental group	Theoretical basis of the intervention	Intervention providers	Number of interventions, frequency and duration of each intervention	Duration of intervention	Sample size(control/experimental group)	Ending indicators	Scale	Outcome
Nyamathi et al., 2013 (31)	United States	Quantitative Research	HIV positive women in rural India	31 (5.3)	Asha-Life (AL) Intervention	The Comprehensive Health Seeking and Coping Paradigm (CHSCP)	expert physicians, nurses, spiritual leaders, and the project director	6 times, 45 min/times		34/34	Internalized HIV Stigma	10-item scale adapted by HSS	The findings of this study reveal that the AL intervention, delivered by lay health women (Asha), significantly reduced internalized stigma
Madona et al., 2024 (47)	Indonesia	Quantitative Research	HIV positive young patients in Indonesia	Mostly between 20 and 24	Pandang Dengar ODHIV campaigns			4 times/week, maximum runtime of 1 min per video	3 weeks	45/45	HIV stigma	Questionnaire developed by the GSDIWG and the Strive Research Alliance to assess HIV stigma in the general population	The Pandang Dengar ODHIV campaign on Instagram significantly reduced HIV stigma among Pati’s youths
Lifson et al., 2023 (32)	United States	Quantitative Research	HIV patients in rural areas of Ethiopia	30	Community support intervention		Community Support Workers	Meet with clients weekly in a community setting for the first 3 months, then 2–4 times per month as clients become more stable		980/819	Internalized HIV Stigma	5-item scale of the HASI-P	Every year after enrollment, scores were lower for intervention compared to control participants. The greatest decrease in the intervention group was from baseline to 12 months, after which there were small incremental increases.
Masquillier et al., 2014 (46)	Belgium	Quantitative Research	HIV patients in South Africa	38.97 (9.34)	Community support intervention		Peer adherence supporters	2 times/week	18 months	630	HIV stigma	10-item scale adapted by HSS	Having a peer adherence supporter has a de-stigmatizing effect on PLWHA
Rongkavilit et al., 2014 (23)	United States	Quantitative Research	Thai HIV positive men who have sex with men	22.5 (2.1)	MI-based Healthy Choices intervention	Motivational interviewing	Psychologist	4 times, 1, 2, 6 and 12 weeks after baseline visit		37/37	HIV stigma	12-item scale adapted by HSS	There was no significant difference in HIV stigma scores between the two groups.
Psaros et al., 2022 (36)	United States	Hybridization study	HIV positive perinatal women with depression in Australia	Median: 24	Problem-solving therapy and the LifeSteps adherence intervention	Problem-solving therapy and the LifeSteps adherence intervention	A trained lay counselor	8 times		8/14,	HIV stigma	33-item scale of the HASI-P	There were no significant differences in perceived social support and stigma by condition, though both study arms experienced significant decreases in stigma from baseline to post-treatment.
Nestadt et al., 2019 (37)	United States	Quantitative Research	HIV positive adolescents in Thailand	12.28 (1.41)	SOC + psychosocial intervention	Social Action Theory(SAT)	Social worker	11 times	6 months	43/45	Internalized HIV Stigma	Adaptation of the Pediatric Epilepsy-Related Stigma Scale Derived from Westbrook LE	Participant stigma studies are statistically unable to detect differences in treatment effects
											Externalized HIV Stigma		
Bhatta et al., 2017 (38)	Nepal	Quantitative Research	HIV patients in Nepal	36.1 (7.8)	Standard Care + a social self-value empowerment intervention	Social learning and action theory	Two national level trainers with a public health graduate degree	6 times/week, 90 min/times	6 weeks	66/66	Internalized HIV Stigma	Genberg’s 23-Item HIV Stigma Scale	Stigma was significantly lower in the intervention group, and the estimated difference in stigma improvement between the intervention and control groups at 3 and 6 months from baseline was significant.
Dow et al., 2020 (17)	United States	Quantitative Research	HIV positive adolescent patients in Tanzania	18.1 (2.3)	SOC + Cognitive behavioral therapy (CBT) + Interpersonal psychotherapy (IPT) + Motivational interviewing (MI)	Cognitive behavioral therapy (CBT) + Interpersonal psychotherapy (IPT) + Motivational interviewing (MI)	Led by six team leaders aged between 24 and 30. Half of the team leaders have practical experience and the others have experience in providing mental health interventions for children.	13 times, 90 min/times, every Saturday	4 months	47/58	HIV stigma	10-item scale from HSS Simplified	Both study groups showed a decrease in internalized stigma at 6 months; however, externalized stigma increased in the intervention group and decreased in the SOC group.
											Internalized HIV Stigma		
											Externalized HIV Stigma		
Budhwani et al., 2021 (24)	United States	Quantitative Research	HIV positive adolescent patients in the United States	21.38 (1.86)	A Healthy Choices Intervention Based on Motivational Enhancement Therapy (an Adapted Version of Motivational Interviewing)	Motivational Enhancement Therapy (an Adapted Version of Motivational Interviewing)	Community health workers	4 times	10 weeks	90/93	HIV stigma	HSS simplified 10-item scale	Not only did YLWH who received the Healthy Choices intervention experience reductions in self-reported levels of stigma from pre- to post-intervention, but that these reductions were sustained over time.
Yigit et al., 2021 (26)	United States	Quantitative Research	New HIV patients receiving HIV care in the United States	35.72	Information-motivation-behavioral skills(IMB)intervention	Information-motivation-behavioral skills (IMB)	Research consultant	4 times	12 months	186/185	Internalized HIV Stigma	7-item scale adapted by HSS	Interventions are effective in reducing internalized HIV-related stigma.
Odhiambo et al., 2023 (43)	Kenya	Quantitative Research	HIV positive widows in Kenya	42.8 (8.4)	A multisectoral climate adaptive agricultural livelihood intervention					145	Internalized HIV Stigma, Anticipated HIV stigma, Enacted sitgma	9-item scale of the IA-RSS	Stigma scores were significantly and statistically reduced in the intervention group compared to the control group
			HIV positive married women in Kenya	35.8 (9.0)						232			Stigma scores were significantly and statistically reduced in the intervention group compared to the control group
Tshabalala et al., 2011 (18)	South Africa	Hybridization study	HIV positive women in South Africa		ART+Cognitive-behavioral interventions	Cognitive-behavioral intervention	Author	8 times, 1 time/week	2 months	10/10	Internalized HIV Stigma	Serithi 16-Item Internalized Stigma Scale	The experimental group reported significantly lower scores at post-treatment compared to the control group
											Enacted sitgma		
Luoma et al., 2023 (5)	United States	Quantitative Research	HIV infected individuals who inject drugs in St. Petersburg, Russia	38.1 (5.3)	Usual care +Acceptance and commitment therapy	Acceptance and commitment therapy	Psychologists	3 times/week, 2 h/time	1 month	33/67	Internalized HIV Stigma	The 7-item scale of the IA-RSS	No changes in stigma performance were found for intervention participants
Bryant et al., 2023 (51)	United States	Quantitative Research	Black women infected with HIV in the southern United States	32.15 (7.21)	Entertainment-education intervention			1 time		130	Internalized HIV Stigma	5-item scale adapted from IA-RSS	There was no statistically significant difference in the difference in internalized stigma between participants in the control and intervention groups.
											Anticipated HIV stigma	5-item scale adapted by HSS	There was no statistically significant difference in the difference in internalized stigma between participants in the control and intervention groups.
Walsh et al., 2024 (19)	United States	Quantitative Research	HIV positive older adult(s) in rural areas of the southern United States	58.05 (6.26)	Social support intervention+Supportive Expressive Group Therapy (SEGT) Intervention	Social support intervention+Supportive Expressive Group Therapy (SEGT) Intervention	Psychiatrist	8 times/week, 90 min/times		61	Internalized HIV Stigma	The 6-item Internalized Stigma Subscale of the HIV Stigma Mechanism Scale	Intervention significantly reduced internalized stigma
					Cognitive-behavioral intervention	Cognitive behavioral therapy	Psychiatrist	6 times/week, 90 min/times			Internalized HIV Stigma		Intervention significantly reduced internalized stigma
Denison et al., 2020 (44)	United States	Quantitative Research	HIV positive youth in Zambia	19	Peer group intervention	Social cognitive theory	A health care provider (HCP), their assigned youth peer mentor (YPM), and an adult caregiver (if invited by the youth participant)	1 time/month	6 months	137/136	Internalized HIV Stigma	3-item scale of the IA-RSS	Significant reductions in internalized stigma experienced by intervention group participants across all study sites
Barroso et al., 2014 (52)	United States	Quantitative Research	HIV positive women in the southern United States	45.9	Stigma reduction interventions			Watch the videos at least once a week for the first 4 weeks, then more or less as needed for weeks 5–12.		48/51	Internalized HIV Stigma	28-item scale of the IHSS	A large effect of the intervention in reducing overall stigma was observed compared to the control group.
Derose et al., 2024 (33)	United States	Quantitative Research	HIV patientsin the Dominican Republic	43.5 (11.8)	An Urban Gardens and Peer Nutritional Counseling Intervention		Agronomists, the clinic’s peer counselor, professional nutritionists	3–4 sessions, 30 min/session	7–9 months	63/46	Internalized HIV Stigma	8-item scale adapted from HSS	Participants in the novel urban garden and peer nutrition counseling interventions reduced internalized and experienced stigma compared to the standard of care provided to participants in the control clinic
											Enacted sitgma		
Peltzer et al., 2018 (39)	South Africa	Quantitative Research	HIVpositive women during the perinatal period	28.41 (5.78)	EnhancedIntervention (EI).:standard care plus the ‘Protect Your Family’ intervention		Non-professional health workers	4 prenatal, 2 postnatal		357/342	HIV stigma	HSS	Intervention participants assessed a reduction in all aspects of stigma
Musanje et al., 2024 (29)	Uganda	Quantitative Research	HIV positive adolescents in Uganda	17 (1.59)	SOC + Acceptance and commitment therapy	Acceptance and commitment therapy	Lay providers such as counsellors and adolescent peers	1 time/week, 90 min/times	1 month	61/61	Internalized HIV Stigma	6-item scale of the IA-RSS	The intervention was associated with a significant reduction in stigma, meaning that stigma scores were substantially and statistically significant lower
Mkumba et al., 2020 (20)	United States	Hybridization study	HIV patients aged 12–24 in Tanzania	17.8 (2.35)	Trauma-Informed Cognitive Behavioral Therapy (TI-CBT) + Interpersonal Psychotherapy (IPT) + Motivational Interviewing (MI)	Trauma-Informed Cognitive Behavioral Therapy (TI-CBT) + Interpersonal Psychotherapy (IPT) + Motivational Interviewing (MI)	Groups of 8–11 young people with trained youth leaders	10 times	4 months	47/58	HIV stigma	16-item scale adapted by HSS	Mean total stigma scores were lower in the intervention group than in the control group, and total stigma scores were lower in both the intervention and control groups compared to baseline, but externalized stigma was higher in the intervention group compared to baseline
											Internalized HIV Stigma		
											Externalized HIV Stigma		
Bogart et al., 2022 (13)	United States	Hybridization study	Sexual minority group of HIV positive Latinx men in the United States	52.9 (12.9)	Cognitive-behavioral interventions	Cognitive behavioral therapy	Two trained peer counselors with expertise in Latino SMM group therapy	8 times	4 months	38/38	Internalized HIV Stigma	IA-RSS	Intervention shows small to medium effect sizes on internalized HIV stigma
Bogart et al., 2022 (40)	United States	Quantitative Research	African Americans who are HIV positive in the United States	49.1 (12.4)	Usual Care+Culturally Consistent Adherence Intervention		One black peer counselor	5 times	6 months	123/122	Internalized HIV Stigma	IA-RSS	The effect of the intervention on internalizing HIV stigma was not significant
Williams et al., 2024 (27)	United States	Quantitative Research	HIV positive patients in South Africa	32.28 (9.28)	Expanding Social Network Recruitment to HIV Testing		Patients themselves	1 time		337/97	HIV stigma	Adaptation of the TRIP program scale	E-SNRHT participants experienced significant decreases between baseline and a 6–10 week follow-up in both anticipated HIV-related stigma and enacted HIV-related stigma. E-SNRHT participants experienced larger decreases in enacted HIV-related stigma than did risk network recruitment participants.
Miles et al., 2003 (28)	United States	Quantitative Research	HIV positive African American mother	37	The self-care symptom management intervention		Three nurses (two African American and one white)	6 times	3 months	50/59	HIV stigma	Demi HIV Stigma Scale	Mothers in the intervention group reported less stigma than mothers in the control group 6 months after the end of the intervention.
Echenique et al., 2014 (41)	United States	v	HIV positive older women in the United States		An educational brochure on sexual risk reduction+p- sychoeducationa			4 times/week, 2 h/times		100/200	HIV stigma	HSS	Females in the intervention group were more likely to report a reduction in high risk sexual behavior and in perceived stigma than women in the control group
Hickey et al., 2021 (25)	United States	Quantitative Research	HIV-positive patients in rural western Kenya	34.5 (10.4)	Social support intervention		Group of 5–10 close family members, friends and other members of the social support system	8 times, 1 time/2 weeks	4 months	150/154	Internalized HIV Stigma	Earnshaw HIV Stigma Scale	Intervention reduced stigma (−0.3 units on a 5-point scale, 95% CI -0.40 to −0.17)
											Anticipated HIV stigma		
											Enacted sitgma		
Zhu et al., 2018 (48)	China	Quantitative Research	HIV-infected patients with increased depressive symptoms in Guangzhou, China.	27.5	Cognitive Behavioral Stress Management + Physical Activity Promotion			12 times	3 months	150/150	HIV stigma	14-item scale within HSS	Run4Love mHealth Intervention Significantly Reduces Depressive Symptoms and HIV-Related Stigma at 3-, 6-, and 9-Month Follow-Up Compared to Controls
Shim et al., 2022 (49)	Republic of Korea	Quantitative Research	HIV patients in Korea		The information-motivation-behavioral skills	The information-motivation-behavioral skills			1 month	16/17	HIV stigma	The 6-item scale of the IA-RSS	Over time, the intervention group showed a statistically significant reduction in perceived stigma over the control group
Graff et al., 2023 (30)	United States	Quantitative Research	HIV-positive gay, bisexual and other men who have sex with men in Kenya	29.2	The information-motivation-behavioral skills	The information-motivation-behavioral skills	Health care providers (clinicians and consultants)			33/27	HIV stigma	12-item scale from HSS simplified	Intervention not associated with significant changes in HIV stigma
Willis et al., 2019 (34)	Zimbabwe	Quantitative Research	HIV-infected adolescents in rural Zimbabwe		Standard of care+community adolescent treatmen		Community Treatment Supporters for Adolescents	1 time/week		47/47	HIV stigma		The intervention group reported a decrease in stigma, although not statistically significant (*p* = 0.848). However, the control group showed a statistically significant increase in stigma levels (*p* = 0.01).
Nabunya et al., 2024 (21)	United States	Quantitative Research	Uganda HIV-infected adolescents (10–14 years old)	12.21 (1.41)	Usual Care+the group cognitive behavioral therapy(G-CBT)	Cognitive behavioral therapy	Two health assistant counselors	10 times, 1 time/2 weeks, 1 h/time	5 months	(1) Usual care (n = 29) (2); G-CBT (n = 26); or (3) MFG-FS (n = 34)	HIV stigma	9-item scale of HSS	The intervention had a significant effect on both forms of stigma compared to usual care. Adolescents participating in the MFG-FS intervention reported significantly lower levels of internalized stigma at 3 months compared with usual care. On the other hand, participants in the G-CBT intervention reported lower levels of anticipated stigma at the 6-month follow-up compared to usual care
											Anticipated HIV stigma		
					Usual Care+ the multiple family group-based family strengthening intervention(MFG-FS)		Two parents	1 time/2 weeks, 1 h/time	5 months		Internalized HIV Stigma		
											Anticipated HIV stigma		
Abbas et al., 2023 (22)	Pakistan	Quantitative Research	HIV patients in Pakistan	31.9 (8.45)	Art+Cognitive Behavioral Therapy	Cognitive Behavioral Therapy		8 times		63/63	HIV stigma	HSS	Significant mean score differences were found between the intervention group and the waitlist control group on the HIV Stigma Scale, suggesting that CBT plays an important role in addressing HIV stigma.
Roopal J. Singh,2020 (35)	India	Quantitative Research	HIV-positive drinking men in India	43	A multilevel approach to interventio	Social ecological theory				188/188/188/188	HIV stigma	16-item scale adapted from HSS	Cycle 1: All three interventions effectively reduced overall stigma and improved personalized stigma, negative self-image, and public attitude concerns. Disclosure concerns were also successfully addressed, allowing policymakers to select the most suitable approach based on needs. Cycle 2: No further reduction in stigma scores was observed across intervention combinations, except for a slight increase in negative self-image in the GI + CA group.
													However, the improvements from Cycle 1 were largely sustained, while stigma worsened in the control group. Cycle 3: The impact of intervention sequences varied significantly: CA + IC + GI sequence: Showed the greatest reduction in stigma, with significant improvements across all four subdomains. GI + CA + IC sequence: Achieved substantial stigma reduction overall and in three subdomains. IC + GI + CA sequence: Initially effective in Cycles 1 and 2, but effects reversed in Cycle 3, ending with no significant difference from the control group.
Rhodes et al.,2022 (50)	United States	Quantitative Research	HIV-Positive GBMSM and Transgender Women in the United States	26 (4.3)	Mobile health interventions	Social Cognitive Theory + Empowerment Theory	weCare Network Health Educators: Trained Interventionists		12 months	98/100	HIV stigma	10-item scale adapted from HSS	Compared to usual care participants, weCare participants did not show significant improvements in HIV stigma
Kaai et al.,2012 (42)	Canada	Quantitative Research	HIV-positive adults not on ART in Kenya	37.4 (7.9)	Modified directly observed therapy	Directly observed therapy	Nurses	2 times/week	6 months	117/117	HIV stigma	16-item scale adapted from HSS	The results suggest that well-managed clinic-based m-DOT does not increase perceived HIV-related stigma.
P hiri et al., 2019 (45)	Zambia	Quantitative Research	HIV-positive mothers in Zambia		Umoyo mother-infant pair model		Staff of health institutions	1 time/month	12 months	14/14	HIV stigma	28-item scale adapted from HASI-P	Umoyo MIP clinics may have had a statistically significant impact on improving social support and reducing HCW (healthcare worker) stigma over time, but did not have any impact on reducing internalized stigma or enacted stigma.
Van Tam et al., 2012 (53)	Sweden	Quantitative Research	People living with HIV in Vietnam	Mostly under 35 years of age	Standard care+Peer Support		Trained HIV-infected persons receiving ART treatment	Visits were conducted once in the first 2 months, and after 2 months, visits were reduced to weekly (if treatment adherence was good) or became more frequent (if adherence was poor).	12 months	109/119	Internalized HIV Stigma	IA-RSS	There were no differences in internal AIDS-related stigma between intervention and control groups or between groups at different clinical stages. Intervention had no effect on internal AIDS-related stigma

### 5.1 General information of the included studies

All 39 included studies were published between 2003 and 2024, with 24 (62%) originating from the United States, 2 (5%) from South Africa, 1 (3%) from China, 1 (3%) from Indonesia, 1 (3%) from Belgium, 1 (3%) from Nepal, 1 (3%) from Kenya, 1 (3%) from Uganda, 1 (3%) from South Korea, 1 (3%) from Zimbabwe, 1 (3%) from Pakistan, 1 (3%) from India, 1 (3%) from Canada, 1 (3%) from Zambia, 1 (3%) from Sweden. All 39 studies were randomized controlled trials. The sample sizes ranged from 22 to 1799 across the studies, totaling 9,058.

Nationalities of the study samples: 10 studies (26%) were from the United States, 4 studies (10%) from South Africa, 4 studies (10%) from Kenya, 2 studies (5%) from Tanzania, 2 studies (5%) from India, 2 studies (5%) from Thailand, 2 studies (5%) from Zambia, 2 studies (5%) from Uganda, 1 study (3%) from Indonesia, 1 study (3%) from Ethiopia, 1 study (3%) from Australia, 1 study (3%) from Nepal, 1 study (3%) from Russia, 1 study (3%) from the Dominican Republic, 1 study (3%) from South Korea, 1 study (3%) from Zimbabwe, 1 study (3%) from Pakistan, and 1 study (3%) from Vietnam.

All samples included in the studies were individuals infected with HIV: 10 studies (26%) involved female HIV-infected populations, among which 2 studies (5%) focused on perinatal women, 2 studies (5%) on mothers, and 1 study on married and widowed women; 2 studies (5%) involved Black women; 8 studies (21%) involved adolescents; 4 studies (10%) targeted sexual minority groups; 1 study (3%) involved older adult(s); 1 study (3%) involved drug users; 1 study (3%) involved men who consumed alcohol; 1 study (3%) involved HIV patients who had recently initiated HIV care; 1 study (3%) involved HIV patients not receiving ART; 1 study (3%) involved HIV patients with depression.

### 5.2 Intervention contents for reducing stigma among people living with HIV

The 22 studies included in this paper explicitly reported the methods/theories upon which HIV-related stigma reduction interventions were based, with five studies employing two or more methods/theories. The most frequently used method was Cognitive Behavioral Therapy (n = 7) (13, 17–22), followed by Motivational Interviewing (n = 4) (17, 20, 23–25), Information-Motivation-Behavioral Skills (IMB) intervention (n = 3) (26–28), and Acceptance and Commitment Therapy (n = 2) (5, 29).

### 5.3 Intervention formats for reducing stigma among people living with HIV

The intervention measures in different studies can be categorized into online and offline approaches, with some studies employing a combination of both (30). Offline interventions are primarily conducted through face-to-face interactions, including counseling (23, 24, 31–35), education (5, 13, 17, 18, 20–22, 25, 26, 29, 32, 33, 36–42), and discussion sessions (13, 17, 18, 21, 24, 26, 28, 30, 37, 38, 43–45). Additional offline strategies include follow-up monitoring of participants to improve adherence (30, 34, 35, 42, 46), educating network members after offline recruitment (27), and providing economic support for participants to purchase agricultural supplies (43). Online interventions are mainly implemented via platforms such as telephone calls, text messages, and social media software. These include delivering educational courses through online platforms (19), posting relevant information or engaging in communication via social media (30, 47–50), and using electronic devices to watch specially produced videos (51, 52).

### 5.4 Implementers of interventions to reduce stigma among people living with HIV

Because online interventions are delivered through electronic devices, only offline interventions involve implementers. Implementers can be broadly categorized into healthcare workers and non-healthcare workers. Healthcare workers include physicians, nurses (28, 31, 42), psychiatrists (19), national trainers with a graduate degree in public health (38), and staff members of health organizations (24, 30, 44, 45); non-healthcare workers include psychologists (5, 23), community support workers (32, 37), researchers (18), patients (20), family members, peers within research advisory groups and social support systems (13, 20, 21, 23, 25, 26, 29, 33, 34, 36, 40, 46), and other individuals living with HIV (17, 53).

### 5.5 Control group interventions in research on reducing stigma among people living with HIV

All studies included in this review were RCTs and had control groups. Compared with the intervention groups, participants in the control groups engaged in various activities, including receiving standard HIV treatment and care (5, 13, 17, 20–26, 28–35, 37–40, 42–46, 50–53); being assigned to a waitlist and later receiving the same intervention as the experimental group (18); recruiting network members offline without providing education; receiving usual care plus a booklet containing standard information (25, 49); receiving general HIV information via software (47); receiving general health education (23) and self-management education (21); and one study did not report the intervention details for the control group (19).

### 5.6 Number of interventions, frequency, and duration

Among the 39 included studies, the number of interventions ranged from 1 to 36. The highest intervention frequency was eight times per week, and the duration of each intervention session ranged from 1 min to 2 h. The total duration of the interventions ranged from 3 weeks to 18 months, with 16 studies not reporting the intervention duration.

### 5.7 Outcome measures and assessment tools

The primary outcome measure was HIV-related stigma, including total stigma, internalized stigma, anticipated stigma, enacted stigma, and external stigma. The range of outcome measurement tools used in the included studies was broad, including the HIV Stigma Scale (HSS)(36, 43, 50); a 14-item subscale of the HSS (48); a modified 16-item scale derived from the HSS (35, 42); a 12-item scale adapted or simplified from the HSS (23, 30); a 10-item scale adapted or simplified from the HSS (17, 20, 24, 31, 46, 50); a 9-item scale from the HSS (21); a modified 7-item scale based on the HSS (26); an 8-item scale adapted from the HSS (33); a 5-item scale adapted from the HSS (51); the Internalized AIDS-Related Stigma Scale (IA-RSS) (13, 40, 51, 53); a 9-item version of the IA-RSS (43); a 7-item version of the IA-RSS (5); a 6-item version of the IA-RSS (29, 49); a modified 5-item version of the IA-RSS (51); a 3-item version of the IA-RSS (44); the 33-item HIV/AIDS Stigma Instrument—Patient Version (HASI-P) (36); a modified 28-item version of the HASI-P (45); a 5-item version of the HASI-P (32); the 28-item Internalized HIV Stigma Scale (IHSS) (52); a questionnaire developed by the Global Stigma and Discrimination Indicators Working Group (GSDIWG) and the Strive Research Consortium to assess HIV stigma in the general population (47); the 23-item HIV Stigma Scale developed by Genberg et al. (38); a modification of the Westbrook LE Pediatric Epilepsy-Related Stigma Scale (37); Serithi’s 16-item Internalized Stigma Scale (18); the six-item internalized stigma subscale from the Mechanisms of HIV Stigma Scale (19); the Demi HIV Stigma Scale (28); the HIV Stigma Scale developed by Earnshaw et al. (25); one study measured HIV stigma using a modified TRIP project-based scale (27); and one study did not specify the name of the questionnaire (34).

### 5.8 Follow-up time

Among the 39 included studies, the follow-up time ranged from 3 weeks to 36 months, with the most common follow-up period being 6 months (n = 10). One study conducted assessments immediately after the intervention; one study conducted follow-up at 12 months postpartum; and another study followed up participants 80 to 120 days after the last intervention. All included studies reported changes after the intervention in their follow-up assessments.

### 5.9 Intervention effect

59% (23/39) of the studies reported significant changes in stigma after the intervention, indicating the effectiveness of the interventions in reducing HIV-related stigma. Two studies reported a significant decrease in stigma after the intervention, but there was no significant difference compared with the control group (36, 47). 28% (11/39) of the studies reported that the intervention had no effect on reducing HIV stigma. One study reported that both the intervention and control groups experienced reduced internalized stigma, but the intervention group showed increased externalized stigma (17). One study reported that the average total stigma score in the intervention group was lower than that in the control group; both groups demonstrated decreased total stigma scores compared with baseline, but the intervention group exhibited increased externalized stigma compared with baseline (20). Additionally, one study using a multilevel intervention achieved different outcomes by modifying the sequence of interventions (35).

## 6 Discussion

Through a scoping review of 39 randomized controlled trials, it was found that although there were significant differences between populations and outcomes, most interventions showed effectiveness in reducing HIV related stigma, particularly those based on cognitive behavioral therapy.

### 6.1 Principal findings

The 39 randomized controlled trials (RCTs) included in this article show that, although overall 59% (23/39) of the studies demonstrated that interventions effectively reduced stigma among people living with HIV, there was substantial variation in intervention effectiveness across different studies.

#### 6.1.1 Standardize intervention content to promote the application and dissemination of interventions

Currently, there is no unified standard for specific intervention programs targeting HIV-related stigma, and the content of interventions varies. In teams conducting HIV stigma interventions, some are composed of healthcare workers while others consist of individuals from social networks, aiming to provide more comprehensive and long-term support to patients in order to enhance the effectiveness of interventions. This study found that research employing cognitive behavioral therapy (CBT) yielded relatively positive outcomes, showing significant effects in reducing stigma. CBT is a structured, problem-oriented approach that uses cognitive and behavioral techniques to challenge dysfunctional beliefs (54). CBT helps individuals reduce negative self thoughts, accept themselves, and ultimately lower internal shame through the deconstruction and reconstruction of cognition and behavior. Research indicates that CBT has largely helped address various mental health issues among people living with HIV/AIDS. These findings suggest that combining antiretroviral therapy (ART) with psychological interventions (CBT) is a better treatment option for people living with HIV/AIDS than standard treatment alone (13, 17, 19). Meanwhile, interventions based on Motivational Interviewing (MI) and Acceptance and Commitment Therapy (ACT) have not demonstrated as consistent an effect as CBT. In some studies, MI intervention has effectively reduced HIV related stigma by empowering individuals and helping them build a strong inner world (23); ACT intervention helps HIV infected individuals accept their inner thoughts, coexist peacefully with pain, establish a more stable and objective self-awareness foundation, guide individuals to shift their perception of stigma towards a meaningful life (5), and thus reduce HIV related stigma. However, these two theories have shown different intervention effects in different studies. Through comparison, it is found that the reason for the different intervention results is likely due to the specificity of the study population. Due to the special psychological conditions of HIV positive drug users and HIV positive men who have sex with men in some studies, the intervention effects of MI and ACT are affected (5, 23), ultimately leading to no significant change in the stigma of HIV infected individuals. In comparison, studies with unclear theoretical foundations or overly broad intervention contents were less effective, indicating that a scientific theoretical basis is crucial in interventions aimed at reducing HIV-related stigma (40, 51). Therefore, during the development of intervention programs, strict adherence to or reference of scientific theories or evidence is necessary to formulate rigorous, standardized, and clearly defined intervention plans, thereby advancing the implementation of interventions targeting HIV-related stigma and promoting patient well-being.

#### 6.1.2 Adjust the design of intervention formats to promote diverse applications of intervention forms

In terms of intervention formats, they are mainly divided into online and offline categories. Face-to-face offline interventions (especially group discussions and support groups) have demonstrated significant advantages in improving enacted stigma and internalized stigma. In-person interactions help break the sense of isolation and provide emotional support. Moreover, offline interventions can adjust content and methods promptly according to participants’ facial expressions and body language, facilitating smooth implementation of the intervention (55). Additionally, these interventions can strengthen individuals’ connections with society and reduce sensitivity to external negative evaluations (56). However, loss to follow-up may occur due to issues such as distance and privacy concerns. Conversely, although online interventions (e.g., app notifications, app-based courses) are convenient and offer better privacy, they largely rely on self-directed learning, which demands higher levels of personal initiative. This often significantly increases the likelihood of incomplete interventions and therefore shows limited effectiveness in reducing deep-seated stigma (57). Particularly for online interventions lacking real-time feedback mechanisms, researchers cannot confirm whether participants genuinely adhere strictly to the planned intervention, making reduced engagement more likely to compromise intervention effectiveness. It may also lead to increased loss to follow-up, affecting the accuracy of research outcomes (58). Therefore, in future studies, researchers should attempt to combine both online and offline intervention formats scientifically, integrating the strengths of each approach. It is essential to ensure smooth implementation of the intervention while simultaneously reducing attrition rates during post-intervention follow-ups, thus guaranteeing effective intervention delivery.

#### 6.1.3 Optimize classification of research subjects to promote flexible application of interventions

The manifestations and degrees of stigma vary significantly across different populations. In known RCT studies, interventions among female HIV-infected individuals generally showed better effectiveness, possibly because this population bears a heavier burden of stigma, pays more attention to health issues, actively seeks services, and women are more likely to engage in interventions through family or community relationships. Additionally, programs such as prevention of mother-to-child transmission (PMTCT) often integrate psychological support, enhancing the accessibility of interventions (59, 60). However, the effectiveness of interventions targeting sexual minority groups and drug users is less ideal. Sexual minority groups and drug users may suffer from comorbid mental or psychological disorders, leading to lower acceptance of psychological interventions. Furthermore, these two groups might experience other types of stigma simultaneously, resulting in varying levels of resistance toward interventions and affecting intervention continuity (61, 62). In some studies focusing on adolescent HIV-infected individuals, the psychological development during adolescence is at a unique stage characterized by identity exploration. Their unstable self-esteem and high sensitivity to privacy limit the effectiveness of interventions (63). Therefore, future intervention designs should be based on the characteristics of target populations, conducting more detailed needs analyses according to sources of stigma and personalizing intervention content adjustments, so that intervention strategies can achieve maximum effectiveness for corresponding populations.

#### 6.1.4 Improve the intervention evaluation system, and promote continuous improvement of intervention protocols

Evaluating the effectiveness of stigma interventions helps intervention implementers objectively understand the strengths and weaknesses of these interventions. Over 20 different versions of stigma measurement tools have been used in included studies, most of which were the HIV Stigma Scale (HSS) or its adapted versions. Although these tools have demonstrated reliability and validity to some extent, differences in scale structure, number of items, and scoring methods have led to discrepancies in defining and capturing the concept of “stigma” across studies. For example, some scales focus primarily on internalized stigma while neglecting enacted stigma and externalized stigma, resulting in incomplete evaluation of intervention effects (5). Some scales were adapted for specific regions and therefore lack generalizability (50). Therefore, differences in selected measurement tools may partially obscure the true effectiveness of the interventions. Additionally, previous studies have lacked long-term follow-up investigations, leading to insufficient evaluation of the long-term efficacy of HIV-related stigma interventions. Thus, future research should not only improve the evaluation system for stigma interventions and conduct multidimensional assessments of their effects, but also require researchers to refine intervention protocols based on findings from prior studies and continue conducting multi-center, large-sample, and long-term studies, in order to enhance the effectiveness of stigma interventions among people living with HIV and provide references for stigma interventions among patients with other diseases.

### 6.2 Limitation

Although this study is based on randomized controlled trials (RCTs) and systematically reviews stigma interventions among people living with HIV globally, it still has certain limitations. First, the included studies show significant heterogeneity in intervention content, theoretical basis, implementation methods, intervention frequency, and follow-up duration, making quantitative synthesis analysis impossible; therefore, only descriptive summaries were conducted, which somewhat limits the precision and generalizability of the findings. Second, although most studies used scales developed from the HIV Stigma Scale (HSS), differences exist across versions in structural design, dimension categorization, and measurement focus, potentially affecting comparability of results across studies. Additionally, this review did not search for grey literature such as conference proceedings, thus carrying a risk of publication bias that might overestimate intervention effects. This study did not conduct a formal methodological quality or bias risk assessment on the included studies. This means that although we systematically report the findings and trends of existing research, we cannot determine whether these findings stem from methodological rigor. The majority of included literature was published in English, and coverage of specific populations such as adolescents, older adult(s), transgender people, and drug users was insufficient, limiting the general applicability of the conclusions.

### 6.3 Recommendations for future research and clinical practice

In response to the limitations revealed in this study, future research on HIV-related stigma interventions can be improved and deepened in the following directions. First, standardized measurement tools that have undergone cross-cultural validation should be adopted as much as possible to ensure comparability of intervention effects and accurate interpretation of results. Second, greater emphasis should be placed on the rigorous application of theoretical models during intervention design, strengthening the integration of theory and practice to enhance the targeting and scientific rigor of interventions. Furthermore, future studies should conduct detailed needs assessments based on the specific characteristics of target populations (e.g., age, gender, social identity) to develop more personalized intervention approaches. At the same time, hybrid models combining online and offline components should be further explored to balance confidentiality, accessibility, and interactivity of interventions. Finally, increased attention should be given to populations that are currently underrepresented in research, such as older adults, transgender individuals, and drug users, thereby expanding the applicability of interventions and truly achieving widespread and precise reduction of HIV-related stigma.

## 7 Conclusion

This study, based on the method of scoping review, systematically summarized 39 randomized controlled trials (RCTs) worldwide, and analyzed the content, format, theoretical basis, and effectiveness of interventions targeting HIV-related stigma among people living with HIV. Overall, most interventions were somewhat effective in reducing HIV-related stigma to a certain extent. However, the variability in intervention effects across different populations, intervention formats, and follow-up periods indicates that there is still room for improvement in HIV stigma reduction interventions.

By comparing the intervention measures included in this study, it is found that cognitive-behavioral therapy based intervention measures should be considered a promising model. Moreover, researchers should choose theories that are suitable for different populations and intervene based on them. Adopting standardized measurement tools to standardize the measurement of stigma related to HIV infected individuals; the trial period should be expanded and the follow-up observation period extended to evaluate the sustained effectiveness of the intervention. At the same time, attention should be paid to populations that are currently under covered by research, such as adolescents, the older adult(s), and transgender individuals, and a mixed online and offline intervention model should be explored to adapt intervention measures to more diverse environments and populations, and to improve the universality and accessibility of interventions. Only by continuously optimizing intervention strategies and addressing the limitations of current research can more effective intervention measures be developed to reduce the stigma faced by HIV infected individuals.

## Data Availability

The original contributions presented in the study are included in the article/Supplementary material, further inquiries can be directed to the corresponding author.
