# Supplementary material for: Intervention measures for stigma in HIV patients: a scoping review of randomized controlled trials
Source: Front Public Health. 2025 Nov 5;13:1655870. doi: 10.3389/fpubh.2025.1655870 (PMC12626875; doi:10.3389/fpubh.2025.1655870)
Supplement: Supplementary file 1 [file Table_1.docx]

**Supplementary files 1. Search strategy**

| Databse | Keywords |
| --- | --- |
| PubMed (571) | ((((("HIV"[Mesh]) OR ("HIV Infections"[Mesh])) OR ("Acquired Immunodeficiency Syndrome"[Mesh])) OR ((((((HIV[Title/Abstract]) OR (AIDS[Title/Abstract])) OR (PLWHA[Title/Abstract])) OR (PLWH[Title/Abstract])) OR (human immunodeficiency virus[Title/Abstract])) OR (acquired immunodeficiency syndrome[Title/Abstract]))) AND (("Social Stigma"[Mesh]) OR ((((Social Stigma[Title/Abstract]) OR (Social Stigmas[Title/Abstract])) OR (Stigma, Social[Title/Abstract])) OR (Stigma[Title/Abstract])))) AND (((("Randomized Controlled Trials as Topic"[Mesh]) OR ("Randomized Controlled Trial" [Publication Type])) OR ("Random Allocation"[Mesh])) OR (((Randomized Controlled Trial[Title/Abstract]) OR (randomized[Title/Abstract])) OR (RCT[Title/Abstract]))) |
| Embase (1105) | #1. 'human immunodeficiency virus'/exp OR 'human immunodeficiency virus' OR 'human immunodeficiency'  #2. 'human immunodeficiency virus infection'/exp  #3. 'acquired immune deficiency syndrome'/exp  #4. hiv:ab,ti OR aids:ab,ti OR plwha:ab,ti OR plwh:ab,ti OR 'human immunodeficiency virus':ab,ti OR 'acquired immunodeficiency syndrome':ab,ti  #5. #1 OR #2 OR #3 OR #4  #6. 'social stigma'/exp  #7. 'stigma':ti,ab,kw OR 'social stigma':ti,ab,kw OR 'social stigmas':ti,ab,kw OR 'stigmas, social':ti,ab,kw OR 'stigma, social':ti,ab,kw  #8. #6 OR #7  #9. 'randomized controlled trial'/exp  #10. 'randomization'/exp  #11. 'randomized controlled trial':ab,ti OR 'random allocation':ab,ti OR random*:ab,ti OR rct:ab,ti  #12. #9 OR #10 OR #11  #13. #5 AND #8 AND #12 |
| Cochrane Library (789) | #1. MeSH descriptor: [HlV] explode all trees  #2. MeSH descriptor: [HlV Infections] explode all trees  #3.MeSH descriptor: [Acquired Immunodeficiency Syndrome] explode all trees  #4. (HIV):ti,ab,kw OR (AlDS):ti.ab,kw OR (PLWHA):ti.ab.kw OR (PLWH):ti.ab.kw OR (human immunodeficiency virus):ti,ab,kw OR (human immunodeficiency virus):ti,ab,kw  #5.#1 OR #2 OR #3 OR #4  #6.MeSH descriptor: [Social Stigma] explode all trees  #7.(Social Stigma).ti,ab,kw OR (stigma).ti,ab,kw OR (Stigmas, Social);t,ab,kw OR (Stigma, Social);ti,ab,kw OR (Social Stigmas):ti,ab,kw  #8.#6 OR #7  #9.MeSH descriptor: [Randomized Controlled Trial] explode all trees  #10.MeSH descriptor: [Random Allocation] explode all trees  #11.(Randomized Controlled Trial):ti,ab,kw OR (Random Allocation):ti,ab,kw OR (RcT):ti,ab,kw OR (random*):ti,ab,kw  #12.#9 OR #10 OR #11  #13.#5 AND #8 AND #12 |
| Web of science(1596) | #1.(((((((TS=(HIV)) OR TS= (HIV Infections))) OR TS= (Acquired Immunodeficiency Syndrome)) OR TS=(AIDS)) OR TS=(PLWHA)) OR TS=(PLWH)) OR TS=(human immunodeficiency virus) and Preprint Citation Index (Exclude – Database)  #2.((((TS=(Social Stigma)) OR TS=(Social Stigmas)) OR TS=(Stigmas, Social)) OR TS=(Stigma, Social)) OR TS=(Stigma) and Preprint Citation Index (Exclude – Database)  #3.(((TS=(Randomized Controlled Trial)) OR TS=(Random Allocation )) OR TS=(random*)) OR TS=(RCT) and Preprint Citation Index (Exclude – Database)  #4.#1 AND #2 AND #3 and Preprint Citation Index (Exclude – Database) |
| Scopus(1127) | ((TITLE-ABS-KEY (("hiv" OR "hiv infections" OR "acquired immunodeficiency syndrome" OR "aids" OR "plwha" OR "plwh" oR  "human immunodeficiency virus" )))AND (TITLE-ABS-KEY \|"social stigma" OR "social stigmas" OR "stigmas, social" OR "stigma,social" OR "'stigma" )) AND TITLE-ABS-KEY "randomized controlled trial" OR "random allocation" OR "random*" OR "rct" )) |
